# Adherence to Three Mediterranean Dietary Indexes and All-Cause, Cardiovascular, and Cancer Mortality in an Older Mediterranean Population

**DOI:** 10.3390/nu17182956

**Published:** 2025-09-13

**Authors:** Carolina Ojeda-Belokon, Sandra González-Palacios, Laura María Compañ-Gabucio, Alejandro Oncina-Cánovas, Manuela García-de-la-Hera, Jesús Vioque, Laura Torres-Collado

**Affiliations:** 1 Unidad de Epidemiología de la Nutrición, Departamento de Salud Pública, Historia de la Ciencia y Ginecología, Universidad Miguel Hernández (UMH), 03550 Alicante, Spain; cojeda@umh.es (C.O.-B.); aoncina@umh.es (A.O.-C.);; 2 Instituto de Investigación Sanitaria y Biomédica de Alicante (ISABIAL), 03010 Alicante, Spain; 3 CIBER Epidemiología y Salud Pública (CIBERESP), Instituto de Salud Carlos III, 28029 Madrid, Spain

**Keywords:** Mediterranean diet, dietary indexes, all-cause mortality, cardiovascular disease, cancer

## Abstract

**Background/Objectives**: A higher adherence to the Mediterranean diet (MedDiet) has been associated with a lower risk of death in different populations, but this association has been insufficiently investigated in the elderly Spanish population. In this study, we assess the association between adherence to three MedDiet indexes and all-cause, cardiovascular disease (CVD), and cancer mortality in a population aged 65 years and older in Spain. **Methods:** The population included 903 participants from two population-based surveys. Diet was assessed at baseline by using validated food-frequency questionnaires (FFQ). We calculated scores of adherence to the MedDiet for three indexes: alternate Mediterranean Diet Score (aMED), relative Mediterranean Diet Score (rMED) and 17-item energy-restricted Mediterranean Diet Adherence Screener (erMEDAS). Deaths were ascertained through the National Death Index of Spain and the Mortality Registry in the Valencian Region during a 12 year follow-up period. Cox regression models were used to estimate hazard ratios (HR) and 95% confidence intervals (95% CI), adjusting for relevant confounders. **Results:** During the 12 years of follow-up, 403 deaths occurred: 160 due to CVD and 90 to cancer. Compared to participants in the lowest tertile of adherence to aMED, those in the highest tertile showed a 30% lower risk of all-cause mortality, HR = 0.70 (95% CI 0.51–0.96). In addition, per two-point increase in aMED, we observed a 17% lower risk of all-cause mortality, HR = 0.83 (95% CI 0.73–0.95), and a 21% lower risk of CVD mortality, HR = 0.79 (95% CI 0.64–0.99). A 9% lower risk of all-cause mortality was also observed per two-point increase in the rMED score, HR = 0.91 (95% CI 0.84–0.99). Compared to participants in the lowest tertile of adherence to rMED, those in the highest tertile showed evidence of a marginally significant, lower risk of cancer mortality, HR = 0.55 (95% CI 0.29–1.04). No association was observed between the erMEDAS index and mortality for any cause. **Conclusions:** High adherence to the MedDiet, as measured by aMED and rMED indexes, was associated with lower all-cause, CVD, and cancer mortality in an older Mediterranean population after 12 years of follow-up.

## 1. Introduction

Higher adherence to the Mediterranean diet (MedDiet) has been associated with a decreased risk of all-cause, cardiovascular diseases (CVD), and cancer mortality in different populations [1,2,3,4,5,6]. Particularly in the case of cardiovascular mortality, the evidence is strong [7], but the effect of the traditional MedDiet in older populations and for some other specific causes of death has been insufficiently investigated.

The traditional MedDiet is a dietary pattern characterized by a high consumption of plant-based foods such as vegetables, fruit, whole and minimally processed cereals, virgin olive oil, legumes, and nuts; moderate-to-high consumption of fish and shellfish; low-to-moderate intake of dairy products; low consumption of meat and meat products and moderate consumption of wine during meals [8,9]. The Mediterranean Diet Scale (MDS) was the first dietary index to assess adherence to the MedDiet [10]. Since then, different dietary indexes have been proposed to assess adherence to the MedDiet [11]. Many of these indexes assign scores according to consumption distributions (median or tertiles) of foods or nutrients, such as the alternate Mediterranean Diet Score (aMED) and relative Mediterranean Diet Score (rMED) [12,13], two of the most widely used indexes. However, there are other MedDiet indexes which score positively when the specific criteria are met, such as the 17-item energy-restricted Mediterranean Diet Adherence Screener (erMEDAS) [14], which is based on a qualitative assessment of a list of questions related to foods, dietary preferences, and culinary techniques. The use of indexes to characterize adherence to the MedDiet allows for a direct comparison between the results of different studies and makes associations between diet and risk of disease more consistent.

The aMED index has been shown to have protective effects for many chronic diseases and total mortality [15,16,17,18,19]. In the Nurses’ Health Study (NHS), a higher aMED score was associated with a lower CVD mortality [20] but found no association between aMED score and all-cause or colorectal cancer mortality [19]. Other studies in the Multiethnic Cohort observed an inverse association between a higher aMED score and all-cause, CVD, and cancer mortality [15,17,21]. In another study based on Newfoundland Familial Colorectal Cancer Registry (NFCCR) data, the lowest aMED scores were significantly associated with a higher risk of all-cause mortality [18]. The European Prospective Investigation into Cancer and Nutrition (EPIC) cohort study, showed an association between a high rMED score and a reduction in all-cause and CVD mortality. However, a high rMED score was not significantly associated with cancer mortality [22]. The erMEDAS is a relatively new index with limited accumulated evidence, although a recently published intervention trial has shown a beneficial association between high erMEDAS scores and the cardiometabolic profile [14].

Most recent evidence supports an inverse association between high adherence to the MedDiet and mortality. However, this association between adherence to the MedDiet and all-cause, CVD, and cancer mortality has been less exhaustively investigated in the Mediterranean population aged 65 years and older. Many studies in older populations have evaluated adherence to the MedDiet using MDS [23,24,25,26], while others using aMED [16]. On the other hand, many studies assessed the association between the MedDiet and mortality, mostly in non-Mediterranean populations with a wide age range [13,15,17,21], with specific populations, like female nurses [19,20], or focused on specific cancer sites [18]. Thus, the effect of the MedDiet on long-term mortality in the older Mediterranean population has still not been sufficiently investigated. In this study we aimed to assess the association between adherence to the MedDiet, using three indexes, aMED, rMED, and erMEDAS, and the risk of all-cause, CVD, and cancer mortality in Mediterranean population aged 65 years and older, from two population-based studies.

## 2. Materials and Methods

### 2.1. Study Design and Population

We analyzed the data of participants from two population-based surveys: the European Eye Study (EUREYE), conducted in 2000–2001, and the Valencia Nutrition Survey (VNS) conducted in 1994–1995. Characteristics of both studies have been detailed previously [27,28]. Briefly, EUREYE was a multi-centre, population-based, analytic cross-sectional study with retrospective and current exposure measurements, which included 597 participants aged 65 and above in the province of Alicante in the Valencian Region of Spain. The VNS was a health, nutrition, and examination survey, which included 306 participants, aged 65 and above, who constituted a representative sample of an adult population, from the three provinces of the Valencian Region of Spain. The analysis in the present study was conducted with 903 participants (392 men, 511 women). Ethical approval for both studies was obtained from the Local Ethical Committee of the Hospital of San Juan and Miguel Hernández University, Alicante, Spain (QLK6-CT-1999-02094 for EUREYE and 2013.51.E.CEIE and 2014.16.E.OEP for VNS). In addition, written informed consent was obtained from all participants.

### 2.2. Dietary Intake and MedDiet Indexes

Trained fieldworkers collected dietary intake, using two similar validated semi-quantitative food frequency questionnaires (FFQ); both of them showed satisfactory reproducibility and validity [29,30]. The main difference between the two FFQ was the number of total food items: the EUREYE FFQ collected information about 131 food items while the VNS FFQ collected information about 93 food items. Participants were asked how often they consumed a list of food items on average over the past year, choosing between nine options from “never or less than once per month” to “six or more per day”. Both FFQ collected information about nine food groups: (1) dairy products, (2) eggs, meat, and fish, (3) vegetables and legumes, (4) fruit, (5) bread, cereals, and similar, (6) oils and fats, (7) sweets and pastries, (8) beverages, and (9) processed foods. The daily nutrient intake for each participant was calculated by multiplying the nutrient content of the portion size specified in the FFQ by the reported frequency of intake for each item. Portion sizes used were those standardized for this population, whereas nutrient and energy values were obtained using the US Department of Agriculture food composition tables and other Spanish food composition tables [31,32].

Adherence to the MedDiet for each participant was estimated using aMED, rMED and erMEDAS. The aMED index [12] is a variation of the first MedDiet dietary index published by Trichopoulou in 2003 [10] and was calculated using nine components: fruit (including fruit juices), nuts, vegetables (excluding potatoes), whole-grain products, legumes, fish (including seafood), red and processed meat, alcohol, and the monounsaturated to saturated fat ratio. The components were calculated by the sum of the foods included in Supplementary Table S1. Intakes above the median scored 1 point, and intakes below the median received 0 points, except for red and processed meat, which was scored in reverse. Despite recommendations on alcohol consumption usually differing by sex, this index assigns 1 point for intakes between 5 and 15 g per day, regardless of the participant’s sex, according to the methodology used by Fung et al. [12]. The final score for each participant was the sum of the nine components and ranged from 0 (minimum adherence) to 9 (maximum adherence). The median daily consumption for each food group in the study population was as follows: 2.72 servings for fruit, 0.82 for nuts, 2.97 for vegetables, 0.33 for whole-grain products, 0.28 for legumes, 0.55 for fish, 2.46 for red and processed meat, and 2.46 for the monounsaturated to saturated fat ratio. Since alcohol consumption was evaluated based on a predefined intake range in grams per day, the mean intake among those assigned 0 points was 4.04 g per day, while the mean intake among those who received 1 point was 12.88 g per day. The mean aMED scores by tertile were 1.59 (range 0–2) for the first tertile, 3.42 (range 3–4) for the second, and 5.47 (range 5–8) for the third.

The rMED index [13] is another variation of the original MedDiet dietary index published by Trichopoulou in 2003 [10] and is based on the intake of nine food components: fruit (including nuts and seeds, but excluding fruit juices), vegetables (excluding potatoes), cereals (including white-grain and whole-grain), virgin olive oil, legumes, fish (including seafood), meat (including processed meat), dairy products (including low- and high-fat) and alcohol. The components were calculated by the sum of the foods included in Supplementary Table S1. Each component of rMED was calculated as a function of energy density (grams per 1000 kcal/day) and the score was assigned according to tertiles of components’ intake. The first, second, and third tertiles of intake received scores of 0, 1, and 2, respectively, for all components except for dairy products and meat, which were scored in reverse. Alcohol was considered as a dichotomous variable with the following scores: 2 points for consumptions of 10–50 g/day for men and 5–25 g/day for women, while 0 points were assigned for other consumptions. The final score for each participant was the sum of the nine components and ranged from 0 (minimum adherence) to 18 (maximum adherence). The mean consumption values (in g/day) for the first, second, and third tertiles, respectively, were as follows: for fruit, 86.82, 157.67, and 288.52; for vegetables, 83.16, 154.68, and 255.54; for cereals, 60.68, 94.19, and 139.92; for virgin olive oil, 3.52, 11.12, and 18.36; for legumes, 7.98, 16.09, and 35.06; for fish, 14.39, 27.23, and 50.71; for meat, 25.68, 46.28, and 68.17; and for dairy products, 91.78, 194.56, and 348.33. As alcohol was assessed dichotomously, the mean intake among those assigned 0 points was 2.61 g/day, while those who received 2 points had a mean intake of 16.72 g/day. The mean rMED scores by tertile were 5.76 (range of 0–7) for the first tertile, 8.98 (range of 8–10) for the second tertile, and 11.85 (11–16) for the third tertile.

The erMEDAS index [14] was developed by the PREDIMED-Plus study [33]. This index was calculated according to a 17-point scale based on the following components: extra-virgin olive oil, fruit, vegetables, white bread, whole-grain cereals, meat and meat products, butter and margarine, sugary beverages or sugar-sweetened juices, legumes, fish or shellfish, sweets or pastries, nuts, poultry, “sofrito” sauce, added sugar, non-whole grain cereals, and wine. Compliance with each criteria received a score of 1 point and non-compliance received 0 points. Specific criteria and their cut-offs to assign scores were described in detail previously [14] and in Supplementary Table S2. Since we did not use the “sofrito” item due to a lack of information in the FFQ, the final score for each participant was the sum of the sixteen components and ranged from 0 (minimum adherence) to 16 (maximum adherence).

### 2.3. Assessment of Mortality

Information regarding vital status, date, and the cause of death during the 12 year follow-up period was obtained through the National Death Index from the Spanish Statistical Office and the Mortality Registry in the Valencian Region.

The International Classification of Diseases version 10 (ICD-10) was used to codify the causes of death and to classify them into three categories: CVD (ICD-10: I00-I99), cancer (ICD-10: C00-D49), and all-cause mortality which included CVD, cancer and any other causes of death.

### 2.4. Other Variables

Participants in both studies provided information on socio-demographic, anthropometric, health status, and lifestyle variables. Socio-demographic variables included sex (men, women), age (65–74 years, ≥75 years), study (EUREYE, VNS), and educational level (<primary school, ≥primary school). The anthropometric variable used was waist circumference (I, normal, 78–94 cm in men and 64–80 cm in women, II, moderate, 94–102 cm in men and 80–88 cm in women, and III, large, >102 cm in men and >88 cm in women) [34]. Health status variables included self-reported pre-existing chronic diseases at baseline, diabetes (yes/no), high cholesterol (yes/no), and hypertension (yes/no). Lifestyle variables included total sleeping time (hours per day), television (TV) watching time (hours per day) and smoking status (never, ex-smoker, or current smoker).

### 2.5. Statistical Analysis

A descriptive analysis of socio-demographic variables was performed across the tertiles of adherence to the MedDiet indexes. Categorical variables were described and compared using relative frequencies (%) and Chi-square tests. Continuous variables were described and compared using means, standard deviations (SD) and ANOVA tests. We calculated person-years of follow-up for each participant as the difference between date of baseline interview in each survey and completion date of the 12 year follow-up period or the date of death, depending on which came first. We examined the association between adherence to the MedDiet and risk of mortality at 12 years of follow-up, using Cox proportional regression to estimate adjusted hazard ratios (HR) and 95% confidence intervals (95% CI). Participants were classified according to tertiles of adherence to aMED, rMED and erMEDAS indexes to evaluate the association with all-cause, CVD, and cancer mortality. Finally, we also explored the associations using the scores of the three indexes as continuous variables (per each two-point increase).

We calculated two models: a minimally adjusted model including age and sex, and a multivariable model that included variables identified as potential confounders in the previous literature, as well as those that in the bivariate analysis showed *p*-values < 0.20. The multivariable model was adjusted for age, sex, study, educational level, sleep duration, television watching, smoking status, waist circumference, and self-reported pre-existing chronic diseases (diabetes, high cholesterol, and hypertension).

Likelihood Ratio Test was used to estimate the overall significance of the association between the MedDiet, using the three MedDiet indexes, and all-cause, CVD, and cancer mortality. The *p*-trend was estimated to explore the dose–response relationship for the three indexes used, considering the indexes as continuous variables. Cumulative incidence curves were also generated for tertiles of the three indexes and all-cause mortality to assess the association graphically.

All statistical analyses were performed using STATA software (version 16.1, StataCorp, College Station, TX, USA, http://www.stata.com). The applied statistical tests were bilateral, and statistical significance was established at 0.05.

## 3. Results

Table 1 shows the participants’ baseline characteristics, according to tertiles of adherence to the three Mediterranean dietary indexes. Most participants were female (56.6%), aged 65–74 years (62.8%) and had less than a primary school education (65.1%). The majority also presented with a large waist circumference (66.2%), had never smoked (63.7%), and reported a lower prevalence of self-reported diabetes (80.6%), high cholesterol (79.8%), and hypertension (59.9%). Among the 903 participants, 16.7%, 23.4%, and 27.8% were classified in the third tertile of aMED, rMED, and erMEDAS, respectively. Significant differences were observed by tertiles in several variables, but not uniformly across all scores. For aMED, participants in the highest tertile were more likely to have less than a primary school education (55.6%), a greater proportion did not report high cholesterol (71.3%), and they showed lower mean TV watching time (1.6 h per day). For rMED, participants in the highest tertile were more likely to be women (52.6%), aged 65–74 years (70.1%), and a greater proportion did not report high cholesterol (73.8%). For erMEDAS, participants in the highest tertile were more likely to be women (67.7%), never smokers (71.7%) and a greater proportion did not report diabetes (74.4%).

**Figure 1:**
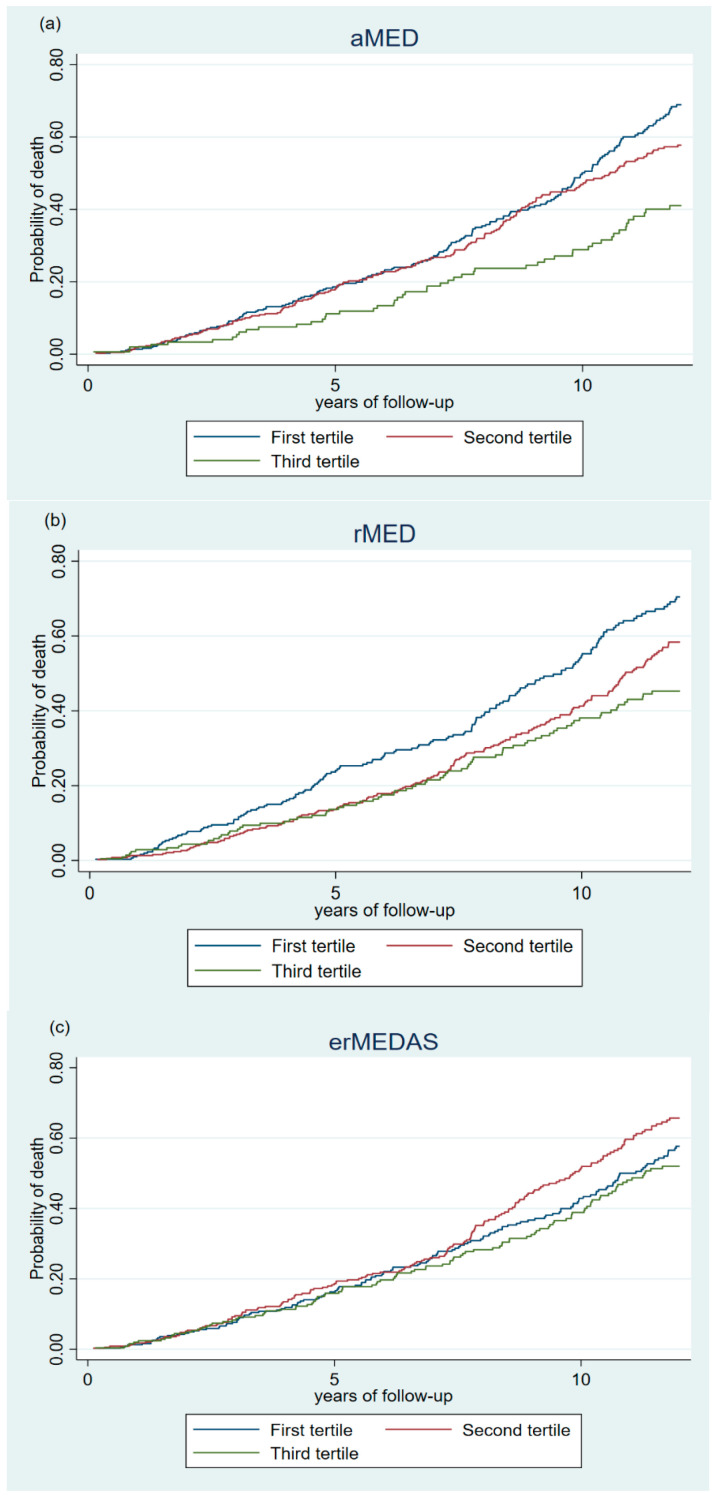
Curves of cumulative incidence for all-cause mortality during the study period according to tertiles of alternate Mediterranean Diet Score (aMED) (**a**), relative Mediterranean Diet Score (rMED) (**b**), and 17-item energy-restricted Mediterranean Diet Adherence Screener (erMEDAS) (**c**), among study participants (n = 903).

Figure 1 shows the cumulative incidence curves for all-cause mortality during the study period, according to tertiles of adherence for the three MedDiet indexes. A lower cumulative incidence of mortality was observed in the highest tertile for all three indexes. For aMED index, a lower incidence was observed throughout the follow-up period, while for rMED and erMEDAS indexes, a lower incidence was observed in the second half of the follow-up period.

Table 2 shows the HR for all-cause, CVD, and cancer mortality according to tertiles of adherence to aMED, rMED and erMEDAS, and for the two-point increase in the score of each index. During the 12 year follow-up period, 8650.0 person-years were accumulated, and 403 deaths occurred, of which 160 (39.7%) were due to CVD and 90 (22.3%) to cancer. In multivariable analysis, a lower risk of mortality for all causes was observed among participants with higher adherence to aMED and rMED.

Compared with those classified in the first tertile of aMED, participants classified in the highest tertile had a 30% lower risk of all-cause mortality, HR = 0.70 (95% CI: 0.51–0.96), with a significant dose–response trend (*p*-trend = 0.008), and the risk of all-cause mortality decreased by 17% per two-point increase in aMED score, HR = 0.83 (95% CI: 0.73–0.95) (Table 2). Regarding the risk of CVD mortality, we observed evidence of an inverse association with the aMED index. Compared with participants in the first tertile of aMED, participants in the highest tertile showed a 40% lower risk of CVD death, HR = 0.60 (95% CI: 0.35–1.02; *p*-trend = 0.037), and the risk of CVD death decreased by 21% per two-point increase in aMED score, HR = 0.79 (95% CI: 0.64–0.99). No significant association was found between aMED and cancer mortality.

For the rMED index, the participants classified in the highest tertile had a 24% lower risk of all-cause mortality, HR = 0.76 (95% CI: 0.57–1.01), compared with those classified in the lowest tertile (*p*-trend = 0.028), and the risk of all-cause mortality decreased by 9% per two-point increase in rMED score, HR = 0.91 (95% CI: 0.84–0.99). No significant association was found between rMED and CVD mortality. Compared to the lowest tertile of adherence, those in the highest tertile showed evidence of a lower risk of cancer mortality, HR = 0.55 (95% CI: 0.29–1.04), *p*-trend = 0.082. There was also evidence of a marginally significant lower risk of cancer mortality for every two-point increase in rMED score, HR = 0.85 (95% CI: 0.72–1.02). Both results slightly approach statistical significance (Table 2).

No significant association was found between erMEDAS and mortality for any cause.

## 4. Discussion

This study shows that a higher adherence to the MedDiet as measured by the aMED and rMED indexes is associated with a lower risk of all-cause mortality after a 12 year follow-up period in a Mediterranean population aged 65 years and older. A higher adherence to the aMED index was also associated with a lower risk of CVD mortality. A lower risk of cancer mortality was observed only for a higher adherence to the rMED index. However, we did not find an association between the erMEDAS index and mortality for any cause.

Our findings are consistent with those from a meta-analysis of 18 studies, in which a two-point increase in several MedDiet indexes was associated with an 8% reduction in the risk of all-cause mortality [35]. Similar inverse associations have also been found between adherence to the MedDiet and all-cause mortality in prospective studies carried out in populations from different continents, in countries like the US [36,37], Iran [38], and China [39]. In the National Health and Nutrition Examination Survey (NHANES), which included 23,212 participants, the lowest tertile of aMED was associated with a 42% increased risk of all-cause mortality [36]. Similarly, the NHS and the Health Professionals Follow-Up Study (HPFS) found a 16% reduced risk in all-cause mortality for participants in the highest aMED quintile, in a population of more than 70,000 participants [37]. In the same line, the Golestan Cohort, with 50,045 participants, found a 20% reduction in risk of all-cause mortality among participants in the highest aMED quintile [38]. In addition, the Singapore Chinese Health Study (SCHS) cohort of 63,257 participants, found a 20% lower risk of all-cause mortality in the highest aMED quintile [39]. Our rMED results are also in line with a previous study conducted in the EPIC cohort with 41,438 participants, which found a 29% lower risk of all-cause mortality for participants in the third tertile of rMED. Furthermore, for each two-point increase in rMED, the risk of all-cause mortality decreased by 6% [22]. Regarding other MedDiet indexes, the PREDIMED study with 7447 participants found a 53% lower risk of all-cause mortality in participants classified in the third tertile of the 14-point Mediterranean Diet Adherence Screener (MEDAS) [40]. The MEDAS index was designed before the erMEDAS index, which is based on an energy-restricted diet, and both showed different associations with mortality. Thus, our study results, based on an older Mediterranean population after 12 years of follow-up, are consistent with these studies, which are mainly based on adult populations from non-Mediterranean areas, aged 26 to 80, and with follow-up periods ranging from 7 to 17 years.

The inverse association we found between higher adherence to the MedDiet and CVD mortality is in line with the results of a previous meta-analysis, based on 13 cohorts that reported that higher adherence to several MedDiet indexes was associated with a 25% reduced risk of CVD mortality [2]. This is consistent with other studies carried out in populations in the US that described an inverse association between the highest quantiles of aMED and CVD mortality [41,42]. The National Institutes of Health-American Association of Retired Persons (NIH-AARP) Diet and Health Study, with over 400,000 participants, found a 20% lower risk of CVD mortality in participants in the fifth aMED quintile [41]. In the same line, in the Women’s Health Initiative Observational Study (WHI OS), a cohort of 93,676 participants showed a 21% reduction in CVD mortality among participants in the fifth aMED quintile [42]. Another study based on the Swedish Mammography Cohort, with 38,428 participants, found a 35% lower risk of CVD mortality in the participants classified in the third tertile of the modified Mediterranean diet score [43]. The division of participants into tertiles, which resulted in a low number of CVD deaths, particularly in the third tertile (n = 18), may have reduced the statistical power to detect significant associations in our study. Despite some associations between aMED and CVD mortality being statistically significant, they were based on a low number of deaths and, therefore, should be interpreted with caution. Our findings regarding CVD mortality are consistent with these studies, which are mainly based on adult populations from non-Mediterranean areas, aged 58 to 79, with follow-up periods ranging from 13 to 17 years.

We found an inverse association between a high adherence to the rMED index and cancer mortality in the less adjusted model. When we included the other covariables in the model, the association was marginally significant, in line with the results of other studies [22]. Regarding aMED, our results are consistent with previous studies that did not find an association between this index and cancer mortality [36,37]. However, other studies found an inverse association between the highest quintile of aMED and cancer mortality [38,39,41,42]. The inconsistency observed in our study for the association between the MedDiet and cancer with respect to others may be due in part to the low number of cancer deaths in our study. Another reason could relate to the inclusion of all cancers as one unique entity, when different cancers may have different risk factors that may counteract among them [44]. Thus, the association between the MedDiet and cancer mortality could not appear when all types of cancer are analyzed together. In a previous review, the MedDiet was associated with a lower risk of several cancers, including stomach, colorectal, breast and upper gastrointestinal and respiratory cancers, but not with other cancer sites [45].

Several biological mechanisms have been proposed to explain why the MedDiet may decrease the risk of mortality. The MedDiet is a dietary pattern rich in antioxidant components such as polyphenols, flavonoids, vitamin E, vitamin C, and beta-carotene [46,47]. These antioxidants are associated with lower oxidative stress, as well as lower risk of atherosclerotic plaque formation, both of which reduce CVD and cancer mortality [46]. In addition, another study found that the MedDiet decreases the risk of mortality because it improves the metabolism of glucose and lipids [48]. The MedDiet may increase HDL cholesterol levels because it contains high levels of monounsaturated fatty acids and low levels of saturated fatty acids, which help to lower cardiovascular risk. Finally, the MedDiet is characterized by a high consumption of virgin olive oil that contains hydroxytyrosol, which has been considered to be a possible mechanism of action against the development of cancer [46].

The lack of association between the erMEDAS index and mortality in our study may be attributed to differences in the scoring system. The aMED and rMED indexes are both based on the distributions of intake of nine similar dietary components within the sample. However, they differ in their approach: aMED assigns binary scores above or below the median for each component, while rMED is based on the tertiles of the score, which reflects the increasing intake levels within the population. Therefore, both indexes are dependent on the sample and provide relative measures of adherence. In contrast, the erMEDAS index comprises 17—16 in our analysis—predefined items with absolute consumption scoring systems, with many based on frequency of use or specific food choices rather than relative population consumption. Whereas aMED and rMED are determined by the quantity consumed relative to the study population, erMEDAS reflects compliance with specific behaviours based on the energy-restricted MedDiet of PREDIMED-Plus trial and includes additional qualitative aspects such as cocking methods and use of certain ingredients, which may not reflect variation in overall diet quality or quantity in this population. Moreover, the score range of erMEDAS is wider, and individual components may have less influence on the total score, which may potentially reduce the index’s sensitivity to detecting associations with mortality. The operational differences are also evident in the observed score distributions: the median aMED scores and rMED scores across tertiles corresponded to distinct ranges of food group intakes, reflecting the stratification of the population, whereas erMEDAS scores were based on a fixed set of criteria that may be less closely aligned with the consumption patterns of this specific regional and age group. In addition, the choice of index can affect the associations between the MedDiet and mortality in prospective cohort studies because different indexes prioritize distinct aspects of the MedDiet and have variable dependence on internal population consumption levels (aMED, rMED) compared to defined criteria (erMEDAS). For example, participants in the same intake category could be assigned differently depending on index-specific cutoffs, leading to potential heterogeneity in risk estimates. This may in part explain why significant associations between MedDiet adherence and mortality were observed with aMED and rMED, but not with erMEDAS in our older Mediterranean sample. A point to be considered is the potential influence on our results of some commonly used dishes like “sofrito”, which is mainly based on vegetables like tomatoes, peppers, and onions cooked with extra virgin olive oil, which were not included in the FFQs. It has been described that some cooking techniques facilitate the availability and absorption of relevant nutrients (e.g., lycopene from tomatoes) [49]. The omission of culturally important food items like “paella” or “sofrito” may have reduced the content validity and discriminatory power of the erMEDAS and attenuated the associations with mortality in our results. This could be a limitation of the FFQ to fully capture different dietary patterns. However, the consumption of main food items included in dishes like “paella” or “sofrito” was collected in our FFQ, likely reducing the possibility of missing relevant associations. Our findings highlight the importance of careful index selection and consideration of score computation methods when interpreting associations between diet and mortality in epidemiological research, particularly in populations with distinct cultural or dietary habits, or specific age profiles.

This suggests that a choice between aMED, rMED and erMEDAS indexes to evaluate adherence to the MedDiet and its health benefits deserves attention. First, both the aMED and rMED indexes have extensive validation and reliability testing in diverse Mediterranean and non-Mediterranean cohorts. Previous studies have demonstrated that these indexes display robust psychometric properties for assessing dietary patterns, including internal consistency, reproducibility over time, and moderate-to-good correlations with biomarker data, as well as reference dietary assessment methods [12,20,21]. Their use of relative, population-based cutoffs (for the aMED, the sample median; for rMED, sample tertiles) enhances their ability to discriminate adherence across the distribution of intake in specific populations, an attribute that supports their construct validity. Furthermore, in our study, the validated FFQ used to calculate the indexes also contribute to measurement accuracy and reproducibility. Second, our data collection was carried out by trained fieldworkers who followed standardized protocols for FFQ administration and data entry. This minimized information bias and maximized the reliability of dietary reported intake, which is particularly crucial when assessing habitual intake in older adults. While the erMEDAS index was developed with strengths such as alignment with the PREDIMED-Plus trial protocol and simplicity for participant use, its scoring system, based on absolute, predefined criteria for 17 individual dietary behaviours, may be less sensitive to variation within Mediterranean older adult populations. In addition, it has comparatively less validation data available, particularly regarding to its psychometric performance in epidemiological studies. Thus, aMED and rMED are especially useful in this context, as supported by the observation of significant associations with mortality outcomes in our analysis, their established validity and reliability in previous research, their sensitivity to differences in intake in a Mediterranean context, and the practicality for computation from standardized FFQ data by trained fieldworkers. These results may have potential implications for the prevention of non-communicable diseases like CVD, by promoting adherence to the MedDiet using the aMED and rMED indexes in nutritional education programmes.

This study has some limitations. Firstly, the dietary information was collected in personal interviews using two validated FFQ at baseline; therefore, we could not evaluate change in dietary patterns throughout the study. However, dietary habits in adulthood usually remain unchanged, and self-reported information about usual diet measured by validated FFQ may be reliable [50,51]. In addition, the FFQ used in VNS (93 items) and EUREYE (131 items) differed in length. Although this may be seen as a limitation for merging responses, the instruments presented the same food group structure, which preserves construct comparability and facilitates harmonization. Secondly, self-reported pre-existing chronic diseases at baseline (diabetes, high cholesterol, and hypertension), can cause a higher risk of mortality, so participants with these diseases may modify their dietary habits as a part of their usual treatment. However, when we carried out the analyses excluding participants with these diseases at baseline, the results remained very similar. Thirdly, we cannot rule out response bias, as participants were volunteers, or misclassification error, as information about diet was self-reported. Although our small sample size could be seen as limiting statistical power, we found significant associations between aMED and rMED indexes and all-cause, CVD, and cancer mortality. As mentioned, the number of cancer deaths was low, which may be a limitation to finding associations. Fourthly, another potential limitation is that we pooled two cohorts, recruited 5–6 years apart. Although we stratified the models by study, it is possible that some heterogeneity or residual unmeasured confounding exists, due to secular changes in diet, lifestyle, or medical care during that period. However, both aged populations were selected from the same area and presented similar characteristics. Finally, another limitation is the method used to calculate the aMED and rMED components, as they are based on the consumption patterns of our study population. Consequently, the calculations may differ in other populations, potentially limiting the generalizability of our findings. However, we have provided the median and mean values used in the index calculations, which facilitate comparison with other populations.

This study also has several strengths. To the best of our knowledge, it is the first to explore the association between the MedDiet and mortality in an older Mediterranean population, using aMED, rMED and erMEDAS indexes. Although other studies have previously reported that the MedDiet has a protective effect, a novelty of this study is the use of a well-defined population, based on representative samples of the population aged 65 and older in a Mediterranean area, the Valencian community. Data used in our study can be considered to be of high quality because the information was obtained by trained fieldworkers through structured interviews, using standardized protocols and validated questionnaires. In addition, the 12 year follow-up period allowed us to explore long-term associations, showing that diet may be a predictor of survival at advanced ages. Finally, the significant dose–response found for some associations may provide evidence for causality.

## 5. Conclusions

Higher adherence to aMED and rMED indexes was associated with a lower all-cause, CVD, and cancer mortality after a 12 year follow-up period. Our study provides valuable new evidence that adherence to the MedDiet in older Mediterranean populations may have long-term beneficial effects on survival. Indexes based on relative consumption, such as aMED and rMED, appeared to be more sensitive in detecting mortality associations in our study than the erMEDAS screener, which is based on more absolute criteria. This underscores the importance of carefully selecting dietary indexes according to the study population and study design. However, more longitudinal studies are needed to confirm our results.

## Figures and Tables

**Table 1:** Socio-demographic and lifestyle characteristics according to aMED, rMED and erMEDAS scores among study participants (n = 903).

	Total	aMED				rMED				**erMEDAS**			
		T1	T2	T3	*p*-Value ^1^	T1	T2	T3	*p*-Value ^1^	**T1**	**T2**	**T3**	***p*** **-Value ^1^**
N (%)	903 (100)	365 (40.4)	387 (42.9)	151 (16.7)		308 (34.1)	384 (42.5)	211 (23.4)		312 (34.6)	340 (37.7)	251 (27.8)	
Score, mean (SD)		1.6 (0.6)	3.4 (0.5)	5.5 (0.7)		5.8 (1.3)	9.0 (0.8)	11.8 (1.1)		3.3 (0.8)	5.4 (0.5)	8.1 (1.2)	
Score, range		0.0–2.0	3.0–4.0	5.0–8.0		0.0–7.0	8.0–10.0	11.0–16.0		1.0–4.0	5.0–6.0	7.0–12.0	
Study, n (%)													
EUREYE	597 (66.1)	235 (64.4)	252 (65.1)	110 (72.9)	0.156	195 (63.3)	261 (68.0)	141 (66.8)	0.424	224 (71.8)	218 (64.1)	155 (61.8)	0.027
VNS	306 (33.9)	130 (35.6)	135 (34.9)	41 (27.2)		113 (36.7)	123 (32.0)	70 (33.2)		88 (28.2)	122 (35.9)	96 (38.3)	
Sex, n (%)													
Men	392 (43.4)	160 (43.8)	170 (43.9)	62 (41.1)	0.815	110 (35.7)	182 (47.4)	100 (47.4)	0.004	154 (49.4)	157 (46.2)	81 (32.3)	<0.001
Women	511 (56.6)	205 (56.2)	217 (56.1)	89 (58.9)		198 (64.3)	202 (52.6)	111 (52.6)		158 (50.6)	183 (53.8)	170 (67.7)	
Age, n (%)													
65–74 years	567 (62.8)	223 (61.1)	245 (63.3)	99 (65.6)	0.610	172 (55.8)	247 (64.3)	148 (70.1)	0.003	197 (63.1)	216 (63.5)	154 (61.4)	0.853
≥75 years	336 (37.2)	142 (38.9)	142 (36.7)	52 (34.4)		136 (44.2)	137 (35.7)	63 (29.9)		115 (36.9)	124 (36.5)	97 (38.7)	
Educational level, n (%)													
<Primary school	588 (65.1)	240 (65.8)	264 (68.2)	84 (55.6)	0.021	198 (64.3)	251 (65.4)	139 (65.9)	0.924	201 (64.4)	225 (66.2)	162 (64.5)	0.873
≥Primary school	315 (34.9)	125 (34.3)	123 (31.8)	67 (44.4)		110 (35.7)	133 (34.6)	72 (34.1)		111 (35.6)	115 (33.8)	89 (35.5)	
Waist circumference (cm) ^2^, n (%)													
I	97 (10.9)	36 (10.0)	40 (10.5)	21 (14.0)	0.624	27 (9.0)	47 (12.3)	23 (11.0)	0.551	29 (9.4)	46 (13.8)	22 (8.9)	0.109
II	204 (22.9)	88 (24.5)	83 (21.7)	33 (22.0)		64 (21.3)	90 (23.6)	50 (23.8)		82 (26.5)	69 (20.7)	53 (21.4)	
III	590 (66.2)	235 (65.5)	259 (67.8)	96 (64.0)		209 (69.7)	244 (64.0)	137 (65.2)		199 (64.2)	218 (65.5)	173 (69.8)	
Smoking status, n (%)													
Never	574 (63.7)	230 (63.4)	243 (62.8)	101 (66.9)	0.696	207 (67.4)	240 (62.7)	127 (60.2)	0.129	186 (59.8)	208 (61.4)	180 (71.7)	0.017
Ex-smoker	206 (22.9)	79 (21.8)	93 (24.0)	34 (22.5)		62 (20.2)	84 (21.9)	60 (28.4)		73 (23.5)	84 (24.8)	49 (19.5)	
Current	121 (13.4)	54 (14.9)	51 (13.2)	16 (10.6)		38 (12.4)	59 (15.4)	24 (11.4)		52 (16.7)	47 (13.9)	22 (8.8)	
Diabetes ^3^, n (%)													
Yes	175 (19.4)	58 (15.9)	85 (22.0)	32 (21.2)	0.094	52 (16.9)	78 (20.3)	45 (21.3)	0.388	28 (9.0)	71 (20.9)	76 (30.3)	<0.001
No	727 (80.6)	306 (84.1)	302 (78.0)	119 (78.8)		255 (83.1)	306 (79.7)	166 (78.7)		283 (91.0)	269 (79.1)	175 (69.7)	
High cholesterol ^3^, n (%)													
Yes	181 (20.2)	57 (15.8)	81 (21.1)	43 (28.7)	0.003	48 (15.7)	78 (20.5)	55 (26.2)	0.002	55 (17.7)	62 (18.5)	64 (25.6)	0.042
No	715 (79.8)	305 (84.3)	303 (78.9)	107 (71.3)		257 (84.3)	303 (79.5)	155 (73.8)		256 (82.3)	273 (81.5)	186 (74.4)	
Hypertension ^3^, n (%)													
Yes	359 (40.1)	142 (39.3)	163 (42.5)	54 (36.0)	0.364	121 (39.8)	159 (41.7)	79 (37.6)	0.615	129 (41.8)	121 (36.0)	109 (43.6)	0.138
No	536 (59.9)	219 (60.7)	221 (57.6)	96 (64.0)		183 (60.2)	222 (58.3)	131 (62.4)		180 (58.3)	215 (64.0)	141 (56.4)	
TV watching, h/d, mean, SD	3.8 (2.0)	3.8 (1.9)	3.9 (2.1)	3.4 (1.6)	0.001	3.8 (2.0)	3.9 (2.1)	3.8 (1.8)	0.151	3.9 (1.9)	3.8 (2.0)	3.7 (1.9)	0.585
Sleeping time, h/d mean, SD	7.8 (2.0)	7.9 (2.1)	7.8 (2.0)	7.6 (1.9)	0.587	8.0 (2.1)	7.8 (2.0)	7.5 (1.9)	0.141	8.0 (2.0)	7.9 (2.1)	7.5 (1.9)	0.418

Abbreviations: SD, standard deviation; EUREYE, European Eye Study; VNS, Valencia Nutrition Survey; aMED, alternate Mediterranean Diet Score; rMED, relative Mediterranean Diet Score; erMEDAS; 17-item energy-restricted Mediterranean Diet Adherence Screener; TV: television. ^1^ *p*-value from Chi-square test (categorical variables) and ANOVA (continuous variables). ^2^ Waist circumference: I (78–94 cm in men, 64–80 cm in women), II (94–102 cm in men, 80–88 cm in women), III (>102 cm in men, >88 cm in women). ^3^ Self-reported diabetes, high cholesterol, and hypertension.

**Table 2:** Associations between adherence to MedDiet according to aMED, rMED and erMEDAS indexes and all-cause, CVD and cancer mortality among study participants (n = 903).

	T1	T2	T3	*p*-Value ^2^	*p*-Trend ^3^	Per Two-Point Increase
aMED						
All-cause (n, %)	365 (40.4)	387 (42.9)	151 (16.7)			
Deaths, n	182	170	51			
Person-years	3423.7	3676.6	1549.7			
HR (95% CI)						
Age- and sex-adjusted	Ref	0.88 (0.71–1.08)	0.61 (0.45–0.84)	0.002	0.001	0.81 (0.71–0.92)
Multivariable ^1^	Ref	0.87 (0.70–1.08)	0.70 (0.51–0.96)	0.026	0.008	0.83 (0.73–0.95)
CVD (n, %)	256 (38.8)	286 (43.3)	118 (17.9)			
Deaths, n	73	69	18			
Person-years	2689.0	3045.6	1327.4			
HR (95% CI)						
Age- and sex-adjusted	Ref	0.82 (0.60–1.15)	0.49 (0.29–0.82)	0.007	0.007	0.75 (0.61–0.93)
Multivariable ^1^	Ref	0.83 (0.59–1.18)	0.60 (0.35–1.02)	0.060	0.037	0.79 (0.64–0.99)
Cancer (n, %)	218 (36.9)	259 (43.9)	113 (19.2)			
Deaths, n	35	42	13			
Person-years	2419.5	2844.4	1271.4			
HR (95% CI)						
Age- and sex-adjusted	Ref	1.03 (0.66–1.61)	0.70 (0.37–1.32)	0.273	0.118	0.80 (0.61–1.06)
Multivariable ^1^	Ref	1.08 (0.68–1.71)	0.86 (0.45–1.64)	0.642	0.392	0.88 (0.66–1.18)
rMED						
All-cause (n, %)	308 (34.1)	384 (42.5)	211 (23.4)			
Deaths, n	156	170	77			
Person-years	2799.3	3757.5	2093.2			
HR (95% CI)						
Age- and sex-adjusted	Ref	0.80 (0.64–1.00)	0.70 (0.53–0.92)	0.012	0.006	0.90 (0.83–0.97)
Multivariable ^1^	Ref	0.82 (0.65–1.03)	0.76 (0.57–1.01)	0.057	0.028	0.91 (0.84–0.99)
CVD (n, %)	216 (32.7)	278 (42.1)	166 (25.2)			
Deaths, n	64	64	32			
Person-years	2247.9	3004.9	1809.2			
HR (95% CI)						
Age- and sex-adjusted	Ref	0.75 (0.53–1.06)	0.69 (0.45–1.06)	0.099	0.048	0.88 (0.78–1.00)
Multivariable ^1^	Ref	0.79 (0.55–1.13)	0.79 (0.51–1.23)	0.292	0.180	0.91 (0.80–1.04)
Cancer (n, %)	187 (31.7)	255 (43.2)	148 (25.1)			
Deaths, n	35	41	14			
Person-years	2012.5	2816.0	1706.8			
HR (95% CI)						
Age- and sex-adjusted	Ref	0.82 (0.52–1.28)	0.50 (0.27–0.94)	0.030	0.025	0.83 (0.70–0.98)
Multivariable ^1^	Ref	0.85 (0.53–1.36)	0.55 (0.29–1.04)	0.066	0.082	0.85 (0.72–1.02)
erMEDAS						
All-cause (n, %)	312 (34.6)	340 (37.7)	251 (27.8)			
Deaths, n	137	164	102			
Person-years	3007.4	3190.3	2452.2			
HR (95% CI)						
Age- and sex-adjusted	Ref	1.19 (0.95–1.49)	1.00 (0.77–1.30)	0.992	0.977	1.00 (0.91–1.10)
Multivariable ^1^	Ref	1.07 (0.85–1.36)	0.86 (0.65–1.13)	0.280	0.257	0.94 (0.85–1.04)
CVD (n, %)	224 (33.9)	245 (37.1)	191 (28.9)			
Deaths, n	49	69	42			
Person-years	2423.1	2596.1	2042.7			
HR (95% CI)						
Age- and sex-adjusted	Ref	1.40 (0.97–2.02)	1.04 (0.68–1.59)	0.845	0.915	0.99 (0.85–1.16)
Multivariable ^1^	Ref	1.25 (0.85–1.84)	0.84 (0.53–1.31)	0.436	0.242	0.91 (0.77–1.07)
Cancer (n, %)	206 (34.9)	210 (35.6)	174 (29.5)			
Deaths, n	31	34	25			
Person-years	2266.2	2310.4	1958.7			
HR (95% CI)						
Age- and sex-adjusted	Ref	1.18 (0.72–1.92)	1.11 (0.65–1.91)	0.693	0.040	1.02 (0.83–1.26)
Multivariable ^1^	Ref	1.16 (0.70–1.91)	1.07 (0.61–1.87)	0.818	0.982	1.00 (0.80–1.25)

Abbreviations: MedDiet, Mediterranean Diet; aMED, alternate Mediterranean Diet Score; rMED, relative Mediterranean Diet Score; erMEDAS; 17-item energy-restricted Mediterranean Diet Adherence Screener; CVD, cardiovascular disease; HR, hazard ratio; CI, confidence interval; EUREYE, European Eye Study; VNS, Valencia Nutrition Survey. ^1^ Multivariable model adjusted for age (65–74, ≥ 75 years), sex, study (EUREYE, VNS), educational level (<primary school, ≥primary school), sleep duration (h/day), television watching (h/day), smoking habit (current, ex-smoker, and never smoker), waist circumference: I, normal (78–94 cm in men, 64–80 cm in women), II, moderate (94–102 cm in men, 80–88 cm in women), III, large (>102 cm in men, >88 cm in women), self-reported chronic diseases (diabetes, high cholesterol, and hypertension) (yes/no). ^2^ *p*-value from the Likelihood Ratio Test. ^3^ *p*-value from *p*-trend test.

## Data Availability

The data presented in this study are available on request from the corresponding author. The data are not publicly available due to confidentiality and ethical reasons.

## Supplementary Materials

The following supporting information can be downloaded at: https://www.mdpi.com/article/10.3390/nu17182956/s1, Table S1: Description of the Mediterranean Diet indexes aMED and rMED [12,13]; Table S2: Description of the Mediterranean Diet index erMEDAS [14].

## Abbreviations

The following abbreviations are used in this manuscript:

MedDiet	Mediterranean diet
CVD	Cardiovascular disease
FFQ	Food-frequency questionnaires
aMED	Alternate Mediterranean Diet Score
rMED	Relative Mediterranean Diet Score
erMEDAS	17-item energy-restricted Mediterranean Diet Adherence Screener
HR	Hazard ratios
CI	Confidence intervals
MDS	Mediterranean Diet Scale
NHS	Nurses’ Health Study
NFCCR	Newfoundland Familial Colorectal Cancer Registry
EPIC	The European Prospective Investigation into Cancer and Nutrition
EUREYE	The European Eye Study
VNS	Valencia Nutrition Survey
PREDIMED-Plus	PREvención con DIeta MEDiterránea-Plus
ICD-10	International Classification of Diseases version 10
SD	Standard deviations
NHANES	National Health and Nutrition Examination Survey
HPFS	Health Professionals Follow-Up Study
SCHS	Singapore Chinese Health Study
MEDAS	14-point Mediterranean Diet Adherence Screener
NIH-AARP	The National Institutes of Health-American Association of Retired Persons
WHI OS	Women’s Health Initiative Observational Study
