# Supplementary material for: Adherence to Three Mediterranean Dietary Indexes and All-Cause, Cardiovascular, and Cancer Mortality in an Older Mediterranean Population
Source: Nutrients. 2025 Sep 13;17(18):2956. doi: 10.3390/nu17182956 (PMC12472705; doi:10.3390/nu17182956)
Supplement: Supplementary file 1 [file nutrients-17-02956-s001.zip › nutrients-3871540-supplementary.pdf]

Table S1. Description of the Mediterranean Diet indexes aMED and rMED (Fung, 2005; Buckland, 2009).

	Components of indexes	EUREYE FFQ items	VNS FFQ items
aMED (servings)	Fruit	Orange juice; apple; pear; orange; mandarin; pink grapefruit; white grapefruit; banana; white grape; red grape; melon; kiwi; peach, nectarine and apricot; cherry, strawberry and raspberry; olive; pineapple, mango and papaya; lemon; watermelon; fig; dried fruit; fruit in syrup.	Orange, grapefruit and mandarin; orange juice; banana; apple and pear; strawberry; cherry; peach; fig; watermelon and melon; grapes; olive, fruit in syrup.
	Nuts	Nuts.	Nuts.
	Vegetables	Spinach; chards; sprouts; kale; cabbage; cauliflower; lettuce; tomato; onion; spring onion; carrot; pumpkin; green beans; cucumber; zucchini; aubergine; red pepper; green pepper; asparagus; mushrooms; garlic; broccoli; avocado; leek; cress; parsnip; artichoke; celery.	Spinach and chards; sprouts, cabbage and cauliflower; lettuce; tomato; onion; carrot and pumpkin; green beans; aubergine, zucchini and cucumber; pepper; asparagus; mushrooms; garlic.
	Whole-grain products	Whole wheat bread, corn.	Whole wheat bread.
	Legumes	Lentils, chickpeas, beans; peas.	Lentils, chickpeas, beans; peas.
	Fish	Fish sticks; fried fish; boiled, grilled white fish; fresh, canned blue fish; crab, prawn, lobster; mussel, clam, oyster; squid, octopus.	Fried fish; boiled, grilled fish; salted fish; canned fish; clam, mussel and oyster; squid, cuttlefish, octopus; prawn, crab, shrimp, lobster; fish sticks.
	Red and processed meat	Beef; pork; lamb; liver, pate; hamburger; veal; offal; bacon; ham; cured meat.	Beef, pork and lamb; liver; offal; hamburger; cured meat; sausage; pate: bacon.
	Alcohol	Grams of alcohol.	Grams of alcohol.
	Monounsaturated/saturated fat ratio	Olive oil; other oils, sunflower, soy, corn; margarine; butter.	Olive oil; oils (sunflower, soy, corn); margarine; butter; pork lard.
rMED (grams)	Fruit	Apple; pear; orange; mandarin; pink grapefruit; white grapefruit; banana; white grape; red grape; melon; kiwi; peach, nectarine and apricot; cherry, strawberry and raspberry; olive; pineapple, mango and papaya; lemon; watermelon; fig; dried fruit; nuts.	Orange, grapefruit and mandarin; banana; apple and pear; strawberry; cherry; peach; fig; watermelon and melon; grapes; olive; nuts.

Vegetables	Spinach; chards; sprouts; kale; cabbage; cauliflower; lettuce; tomato; onion; spring onion; carrot; pumpkin; green beans; cucumber; zucchini; aubergine; red pepper; green pepper; asparagus; mushrooms; garlic; broccoli; avocado; leek; cress; parsnip; artichoke; celery.	Spinach and chards; sprouts, cabbage and cauliflower; lettuce; tomato; onion; carrot and pumpkin; green beans; aubergine, zucchini and cucumber; pepper; asparagus; mushrooms; garlic.
Cereals	White bread; whole wheat bread; speciality bread; breakfast cereals; rice; pasta; corn.	White bread; whole wheat bread; speciality bread; rice; pasta.
Virgin olive oil	Olive oil.	Olive oil.
Legumes	Lentils, chickpeas, beans; peas.	Lentils, chickpeas, beans; peas.
Fish	Fish sticks; fried fish; boiled, grilled white fish; fresh, canned blue fish; crab, prawn, lobster; mussel, clam, oyster; squid, octopus.	Fried fish; boiled, grilled fish; salted fish; canned fish; clam, mussel and oyster; squid, cuttlefish, octopus; prawn, crab, shrimp, lobster; fish sticks.
Meat	Poultry with skin; skinless poultry; game meat; beef; pork; lamb; liver, pate; hamburger; veal; offal; bacon; ham; cured meat.	Poultry with skin; skinless poultry; game meat; beef, pork and lamb; liver; offal; hamburger; cured meat; sausage; pate: bacon.
Dairy products	Whole milk; semi-skimmed milk; skimmed milk; condensed milk; cream; heavy cream; skimmed yogurt; whole yogurt; semi-cured cheese; cottage cheese; custard; pudding; ice cream; butter.	Whole milk; skimmed milk; condensed milk; yogurt; cottage cheese; cream cheese; semi-cured cheese; custard; ice cream; butter.
Alcohol	Grams of alcohol.	Grams of alcohol.

Abbreviations: aMED, alternate Mediterranean Diet Score; rMED, relative Mediterranean Diet Score; FFQ, food frequency questionnaire; EUREYE, European Eye Study; VNS, Valencia Nutrition Survey.

Table S2. *Description of the Mediterranean Diet index erMEDAS (Schröder H, 2021).*

Questions	Criteria for 1 point
Do you use only extra-virgin olive oil for cooking, salad dressings, and spreads?	Yes
How many fruit units (including natural fruit juices) do you consume per day?	≥3
How many servings of vegetables/garden produce do you consume per day? [1 serving: 200 g (consider side dishes as half a serving)]	≥2 (≥1 portion raw or in a salad)
How many servings of white bread do you consume per day? (1 serving: 75 g)	≤1
How many times per week do you consume whole-grain cereals and pasta?	≥5
How many servings of red meat, hamburgers, or meat products (ham, sausage, etc.) do you consume per week? (1 serving: 100-150 g)	≤1
How many servings of butter, margarine or cream do you consume per week? (1 serving: 12 g)	<1
How many sugary beverages or sugar-sweetened fruit juices do you drink per week?	<1
How many servings of legumes do you consume per week? (1 serving: 150 g)	≥3
How many servings of fish or shellfish do you consume per week? (1 serving: 100-150 g of fish or 4-5 units or 200 g of shellfish)	≥3
How many times per week do you consume commercial sweets or pastries (not homemade), such as cakes, cookies, sponge cake or custard?	<3
How many servings of nuts (including peanuts) do you consume per week? (1 serving: 30 g)	≥3
Do you preferentially consume chicken, turkey or rabbit instead of beef, pork hamburgers or sausages?	Yes
How many times per week do you consume vegetables, pasta, rice or other dishes seasoned with *"sofrito"* (sauce made with tomato and onion, leek or garlic and simmered with olive oil)?	≥2
Do you avoid adding sugar to beverages (coffee, tea)?	Yes
How many times per week do you consume non-whole grain pasta or white rice?	<3
How many glasses of wine do you drink per day? (1 glass: 200 ml)	2-3 for men; 1-2 for women

Abbreviations: erMEDAS, 17-item energy-restricted Mediterranean Diet Adherence Screener.
